# The A2A adenosine receptor regulates the formation of new lymphatic vessels in inflammatory microenvironments

**DOI:** 10.1186/2051-1426-3-S2-P71

**Published:** 2015-11-04

**Authors:** Bertrand Allard, John Stagg

**Affiliations:** 1 CRCHUM, Montreal Cancer Institute, Montreal University, Montréal, PQ, Canada

## 

The formation of new lymphatic vessels, or lymphangiogenesis, is a natural process involved in tissue repair and in the resolution of inflammatory reactions. In some cancers, this process is exploited by the tumor to promote its growth and its metastatic dissemination. Our group recently identified a role for the CD73-adenosine pathway in tumor angiogenesis. As angiogenesis and lymphangiogenesis are similar processes, often occurring in parallel, we suspect that the CD73-adenosine pathway could have a role in the regulation of lymphangiogenesis during inflammatory reactions and in the tumor microenvironment.

To confirm this hypothesis, we used in vivo models of inflammatory lymphangiogenesis induced by the administration of lipopolysaccharide (LPS) or Incomplete Freund's Adjuvant (IFA) in the peritoneal cavity of mice. These two models of peritonitis trigger the formation of new lymphatics in the diaphragm that can be analyzed by flow cytometry. To study tumor lymphangiogenesis, we used a B16F10 melanoma model implanted in the mouse foot pad thereby allowing the analysis of tumor cell dissemination in the popliteal draining lymph node.

Using A2A-deficient mice, our results demonstrate that the A2A receptor is required for the proper formation of new lymphatic vessels following LPS and IFA administration. Using multicolor flow cytometry we were able to show that A2A-deficiency impairs both the formation of new lymphatic capillaries from pre-existing vessels and the transdifferentiation of pro-inflammatory myeloid cells into lymphatic endothelial cells. Furthermore, our results also indicate that CD73-derived adenosine has a minor involvement in this process as CD73-deficient mice only display a slight alteration in the recruitment of lymphatic endothelial cell in the LPS-peritonitis model. In the B16 melanoma model, host-A2A deficiency impaired tumor-associated lymphangiogenesis which resulted in a reduced number of tumor cells disseminating to the tumor-draining lymph node.

Collectively, our results demonstrate that the A2A receptor promotes inflammatory and tumor-associated lymphangiogenesis.

